# A rapid identification method for soft tissue markers of dentofacial deformities based on heatmap regression

**DOI:** 10.1038/s41405-024-00189-5

**Published:** 2024-03-01

**Authors:** Guilong Zhou, Yu Zhang, Jinlong Zhao, Lei Tian, Guang Jia, Qin Ma

**Affiliations:** 1 State Key Laboratory of Oral & Maxillofacial Reconstruction and Regeneration, National Clinical Research Centre for Oral Diseases, Shaanxi Clinical Research Centre for Oral Diseases, Department of Orthognathic Trauma Surgery, The Third Affiliated Hospital of Air Force Medical University, 710032 Xi’an, China; 2 Hospital 987, Joint Logistics Support Force, 721000 Baoji, China; 3 School of Computer Science and Technology, Xidian University, 710071 Xi’an, China; 4 Oral Biomechanics Basic and Clinical Research Innovation Team, 710032 Xi’an, China

**Keywords:** Occupational health, Oral diseases

## Abstract

**Objective:**

The purpose of this study was to construct a facial deformity dataset and a network model based on heatmap regression for the recognition of facial soft tissue landmarks to provide a basis for clinicians to perform cephalometric analysis of soft tissue.

**Materials and methods:**

A 34-point face marker detection model, the Back High-Resolution Network (BHR-Net), was constructed based on the heatmap regression algorithm, and a custom dataset of 1780 facial detection images for orthognathic surgery was collected. The mean normalized error (MNE) and 10% failure rate (FR10%) were used to evaluate the performance of BHR-Net, and a test set of 50 patients was used to verify the accuracy of the landmarks and their measurement indicators. The test results were subsequently validated in 30 patients.

**Results:**

Both the MNE and FR10% of BHR-Net were optimal compared with other models. In the test set (50 patients), the accuracy of the markers excluding the nose root was 86%, and the accuracy of the remaining markers reached 94%. In the model validation (30 patients), using the markers detected by BHR-Net, the diagnostic accuracy of doctors was 100% for Class II and III deformities, 100% for the oral angle plane, and 70% for maxillofacial asymmetric deformities.

**Conclusions:**

BHR-Net, a network model based on heatmap regression, can be used to effectively identify landmarks in maxillofacial multipose images, providing a reliable way for clinicians to perform cephalometric measurements of soft tissue objectively and quickly.

## Introduction

Orthognathic surgery aims to address issues with dental function [1] and facial esthetics and to improve the symmetry and coordination of facial structures. As patients’ esthetic requirements have continuously increased, treatment concepts guided by esthetics have gradually become more common in orthognathic surgery. Normal facial shape is the basis for normal social communication. Patients with dentofacial deformities (DFDs) often have social difficulties and can even suffer from feelings of inferiority and depression [2, 3]. Therefore, orthognathic surgeons should determine the treatment targets of orthodontic and surgical operations based on esthetic evaluation of the degree of dental deformity.

Esthetically evaluating the face primarily depends on the measurement and analysis of soft tissue. The advent of soft tissue cephalometric changed the treatment philosophy of orthognathic surgery to a focus on “the coexistence of harmonious facial features and good function” [4, 5]. This change in philosophy suggests that clinicians should fully consider the morphology of soft tissues when making surgical plans [6, 7]. Analysis of soft tissue morphology and structure is important for evaluating facial esthetics and postoperative effects [5], whereas the quantitative analysis of facial protrusion, the nasolabiomental relationship and lateral soft tissue fullness has more clinical significance in diagnosis, treatment planning and assessment of facial coordination. Currently, the diagnosis of DFD is usually based on cephalometric analysis of lateral X-ray or computed tomography (CT) data [8, 9]. However, orthognathic surgeons can analyze the ratio of face width to face height using only soft tissue images. When doctors evaluate facial esthetics, they usually measure facial data by using a ruler or facial arch depending on their work experience, which is time-consuming, subjective and highly experience dependent. Therefore, clinical work still lacks an objective and rapid assessment method for facial soft tissue.

The rapid development of artificial intelligence (AI) in the medical field offers a possibility for addressing this problem [10]. In deep learning (DL) strategies, convolutional neural networks (CNNs) are widely used in medical image analysis and have good image processing capabilities. Sun et al. used a CNN on the LFW database to achieve a facial recognition accuracy of up to 97.45% [11]. Jeong SH [12] used VGG19’s CNN to assess whether patients needed orthognathic surgery, with an accuracy of 89.3%. They found that the CNN was relatively accurate at determining the outline of soft tissue needed for orthognathic surgery based on images alone. Although VGG networks have shown that increasing network depth affects the final performance of the network to some extent, it consumes more computing resources and uses more parameters, thus resulting in a greater memory footprint. Patcas R noted that facial attractiveness in patients with cleft palate can be objectively assessed using AI [13]. Plastic surgeons have used CNNs to assess sex typing after facial feminization surgery [14] and age changes after rhinoplasty and cosmetic surgery [15]. Horst et al.’s DL-based algorithm predicts 3D soft tissue contours after mandibular extension [16], and its prediction accuracy is greater than that of the mass tensor model; moreover, the error accuracy is within the clinically acceptable range [16]. Oguzhan Topsakal et al., using an open-source 3D deformable software, successfully synthesized 980 3D face model datasets using DL [17]. However, whether the accuracy of these synthetic faces can meet medical requirements requires further research.

Heatmap regression is a mainstream method for facial key point recognition. It has the advantages of intuitive visualization, appropriate model selection and interpretability of results [18–20]. Liu et al. [21], Wan et al. [22, 23], Kumar et al. [24] and Huang et al. [25] have all developed face key point detection methods based on heatmap regression. Jun Wan et al. established a more effective facial shape constraint model by designing soft transform modules and hard transform modules to cooperate with each other in a reference heatmap transformer (RHT). Moreover, through RHT fusion and a multiscale feature fusion module (MSFFM), converted heatmap features can be fused with semantic features learned from original faces to generate more accurate landmark heatmaps and achieve more accurate landmark detection [26]. Seoungyyoon Kang et al. proposed an effective semisupervised face feature detection framework based on a hybrid representation called HybridMatch. These methods reduce quantization errors by using high-resolution one-dimensional heatmap representations and promote fast convergence of semisupervised learning by using low-resolution two-dimensional heatmap representations. Moreover, these methods have achieved excellent performance on open source datasets [27].

Although soft tissue cephalometry plays a very important role in the diagnosis of facial deformities, it has not been widely used in clinical practice due to its cumbersome measurement methods and subjective results. Facial detection technology based on DL has been widely studied. However, due to the privacy of medical data, there are currently no facial deformity datasets specifically for orthognathic surgery research, and no researchers have attempted to construct a network model that can simultaneously detect multiple pose images, such as front, side, smile, and open mouth images, that can be used for facial deformity diagnosis.

**Fig. 1:**
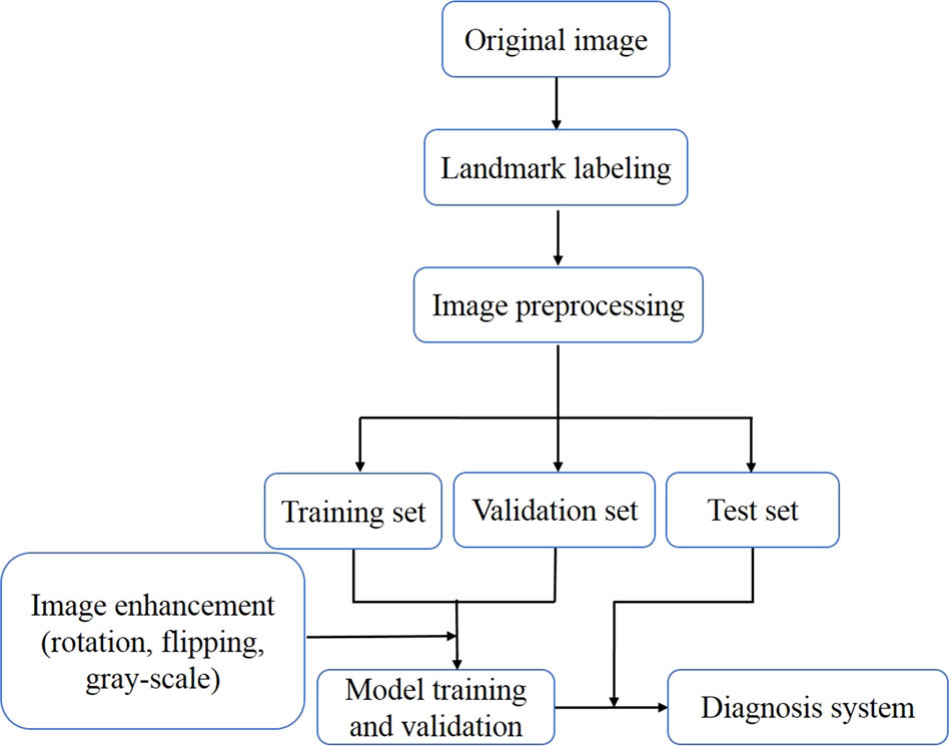
Flowchart of the diagnostic system.

In this study, we successfully collected a dataset of facial developmental deformities that can be used in orthognathic surgery and developed a network model based on a heatmap regression algorithm with a powerful spatial generalization ability that can realize accurate recognition of multiple landmarks in the maxillofacial region. According to these automatically recognized anatomical landmarks, clinicians can objectively obtain facial morphometric indicator data and provide a reliable method for facial soft tissue topography analysis (Fig. 1).

## Materials and methods

### Datasets

The open-source Wider Facial Landmark in the Wild (WFLW) [28] and 300 W [29] face key point datasets were used in this study. The WFLW dataset is a 98-point dataset that is divided into two parts: 7500 faces in the training set and 2500 faces in the test set (full) [28]. The 300 W dataset is a 68-point dataset with a total of 3148 images in the training set and 689 images in the test set (full) [29]. Using 822 facial images of dental deformities used in the study by Jeong SH [12] as a reference and because the open source dataset lacked the DFD and postural images needed for this study, 1030 facial images of patients with maxillofacial deformities who were treated at the Third Affiliated Hospital of Air Force Military Medical University from November 2021 to December 2022 were collected (*n*1 = 1030). The deformities included Class II and Class III bone malocclusions and maxillofacial asymmetric deformities (MADs). In addition, 5 facial postural images were collected from 150 volunteers who were treated from September to December 2022 and who were determined by specialists to not have a history of facial hypoplasia or congenital malformation, infection, trauma, or tumor. These included resting frontal view (RFV), slight mouth opening (SMO), large mouth opening (LMO), postural smile (PS), and resting lateral view (RLV) images, with 750 photos collected in total (*n*2 = 750). The subjects, randomly numbered 1–150, included 105 males and 45 females aged 19–48 years, with a mean age of 27.91 years. A total of 1780 images of DFD patients and volunteers were combined into the custom dataset, which was used as the training set (*n* = *n*1 + *n*2). Using the same inclusion criteria and image acquisition requirements, multipose facial images of another 50 volunteers were collected for the test set (*n*3 = 250). These volunteers included 40 males and 10 females aged 18–39 years, with an average age of 27.98 years. The ratio of the test set to the training set was 14% (n3/n). To test the generalization ability of the network model, photos of the volunteers were taken via mobile phones rather than professional equipment. Because this study involved facial images of volunteers and patients, the datasets generated and/or analyzed during the current study are not publicly available and may be obtained from the corresponding authors upon reasonable request upon successful publication of the paper.

#### RFV

The patient sat in a fixed position, and the overall structure of the face was exposed up to the forehead and back to the auricular region. For these images, the occluded hair was fixed, the eyes looked straight ahead, the line of the pupils was parallel to the ground, the lips were naturally closed, the lower jaw was in a resting position, the nose was in the center of the image, the shoulders were relaxed, the back was straight, and the breathing was gentle. These images are mainly used to assess facial symmetry.

#### SMO

The mouth was open with the incisal edges of the upper and lower central incisors exposed when the mandible was lowered. The images were taken at a distance of 5–20 mm, and the shooting position was the same as that of the RFV images. These images are generally used to evaluate the effect of open-mouth training.

#### LMO

The mouth was open with the mandible lowered to the lowest possible position and the incisal edges of the upper and lower central incisors exposed. The shooting position was the same as that of the RFV images. These images are used to assess joint function or mandibular motor function.

#### PS

This expression is also known as a social smile; it can be reasonably reproduced in daily life through training and does not change with changes in mood. The shooting position was the same as that used for the RFV images. These images are mainly used in smile analysis to evaluate gum exposure, crown ratio and smile arc.

#### RLV

The patient was seated with the body turned 90°, but the position otherwise remained unchanged. The camera height was parallel to the orbital ear plane, and the shooting position was the same as that of the RFV images. These images are mainly used to evaluate the anteroposterior position of the patient’s upper and lower lips and facial soft tissue and the proportional relationship between face height and the size of the mandibular plane angle.

### Ground truth annotations

**Fig. 2:**
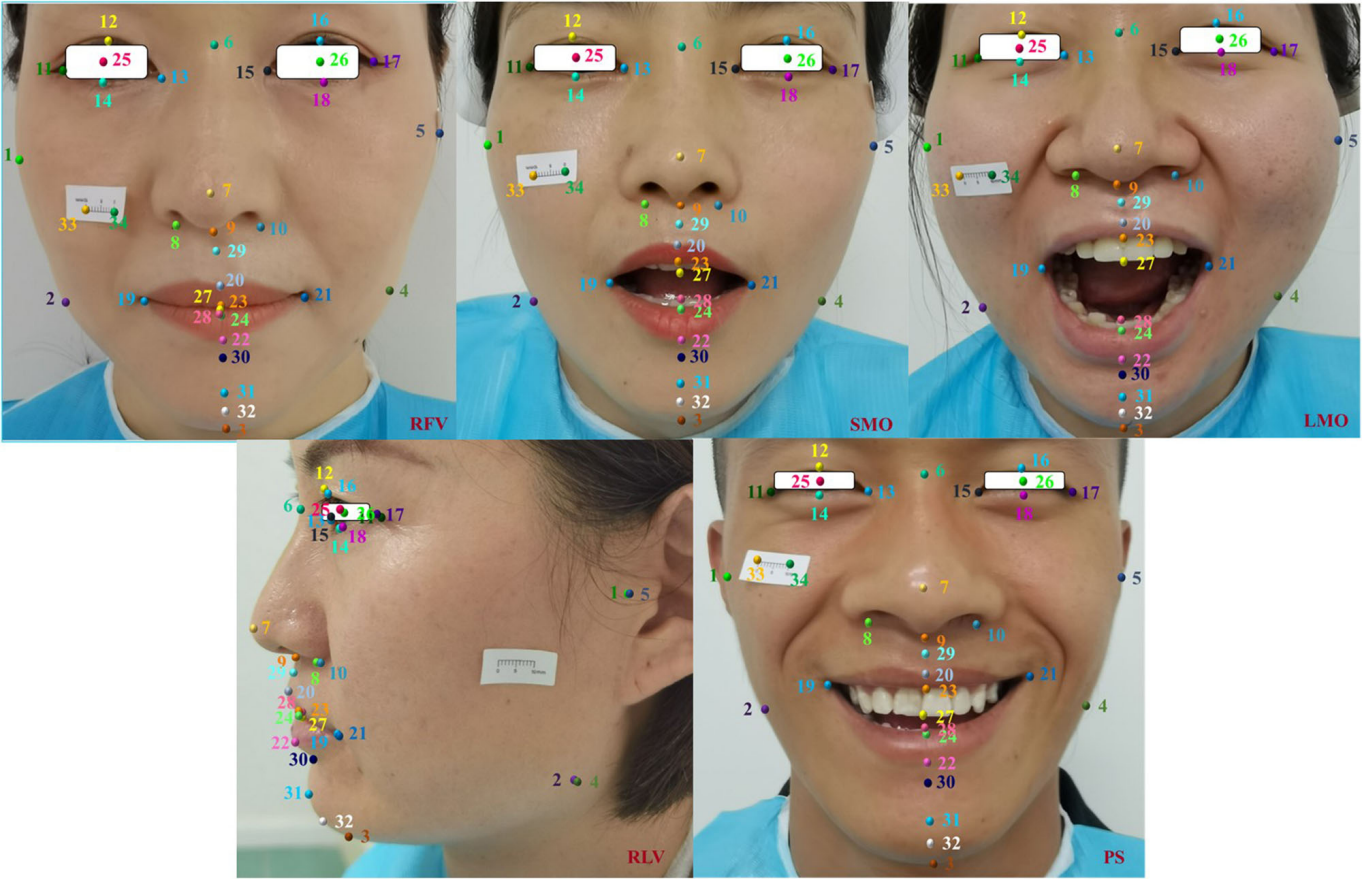
Schematic diagram of the RFV, SMO, LMO, RLV and PS anatomical landmarks. When labeling RLV images, because the contralateral anatomical structure was not visible, the corresponding labels were assigned to the same anatomical position on the visible side.

All the code used in this study was written in the Python 3.8 environment. The Tkinter plug-in was used to develop a marking tool for facial image marks. Three physicians with experience in orthognathic surgery (1 associate chief physician, 1 attending physician, and 1 resident physician) were involved in the process. After unified calibration, image annotation software was used to independently label the training set and test set images 3 times according to the 34 proposed marker definitions (Table 1). To ensure that the distance between subsequent landmarks could be calculated, a 1 cm measuring scale was added to the custom dataset (Fig. 2).

**Table 1:** Definition of anatomical landmarks.

No.	Name	Abbreviation	Definition
1	Right tragus	TR	The midpoint where the right tragus meets the soft tissue of the cheek
2	Right soft gonion	GoR	The most outwards, downwards, and backwards projection of the soft tissue contour of the right mandible
3	Gnathion	Gna	The lowest point of the mental soft tissue in the midsagittal plane
4	Left soft gonion	GoL	The most outwards, downwards, and backwards projection of the soft tissue contour of the left mandible
5	Left tragus	TL	The midpoint where the left tragus meets the soft tissue of the cheek
6	Nasion	N	The midpoint on the soft tissue contour of the base of the nasal root
7	Pronasale	Prn	The most anterior midpoint of the nasal tip
8	Right alar curvature	AcR	The point located at the facial insertion of the right alar base
9	Subnasale	Sn	The midpoint on the nasolabial soft tissue contour between the columella crest and the upper lip
10	Left alar curvature	AcL	The point located at the facial insertion of the left alar base
11	Right exocanthion	ExR	The soft tissue point located at the right outer commissure of each eye fissure
12	Right superior palpebral margin	UPmR	The middle point of the right upper palpebral margin
13	Right endocanthion	EnR	The soft tissue point located at the right inner commissure of each eye fissure
14	Right lower palpebral margin	LPmR	The middle point of the right lower palpebral margin
15	Left endocanthion	EnL	The soft tissue point located at the left inner commissure of each eye fissure
16	Left superior palpebral margin	UPmL	The middle point of the left upper palpebral margin
17	Left exocanthion	ExL	The soft tissue point located at the right outer commissure of each eye fissure
18	Left lower palpebral margin	LPmL	The middle point of the left lower palpebral margin
19	Right cheilion	CR	The point located at the right labial commissure
20	Labiale superius	LS	The midpoint of the vermilion line of the upper lip
21	Left cheilion	CL	The point located at the left labial commissure
22	Labiale inferius	Li	The most inferior point of the upper lip in the midsagittal plane
23	Stomion superius	Sts	The most inferior point of the upper lip in the midsagittal plane
24	Stomion inferius	Sti	The most inferior point of the lower lip in the midsagittal plane
25	Right pupil	PuR	The center of the right pupil
26	Left pupil	PuL	The center of the left pupil
27	Upper incisor	UI	The most mesial point of the crown of the upper central incisor
28	Lower incisor	LI	The most mesial point of the crown of the lower central incisor
29	Subspinale	Ss	The most posterior midpoint of the philtrum
30	Sublabiale	Sl	The most posterior midpoint on the labiomental soft tissue contour that defines the border between the lower lip and the chin
31	Soft pogonion	Pog	The most anterior midpoint of the chin
32	Soft gnathion	Gn	The mental soft tissue at the most anterior and inferior point of the median sagittal direction
33	0		Left end of 1 cm scale
34	1		Right end of 1 cm scale

L stands for left side, R stands for right side.

### Preprocessing and cropping procedure

**Fig. 3:**
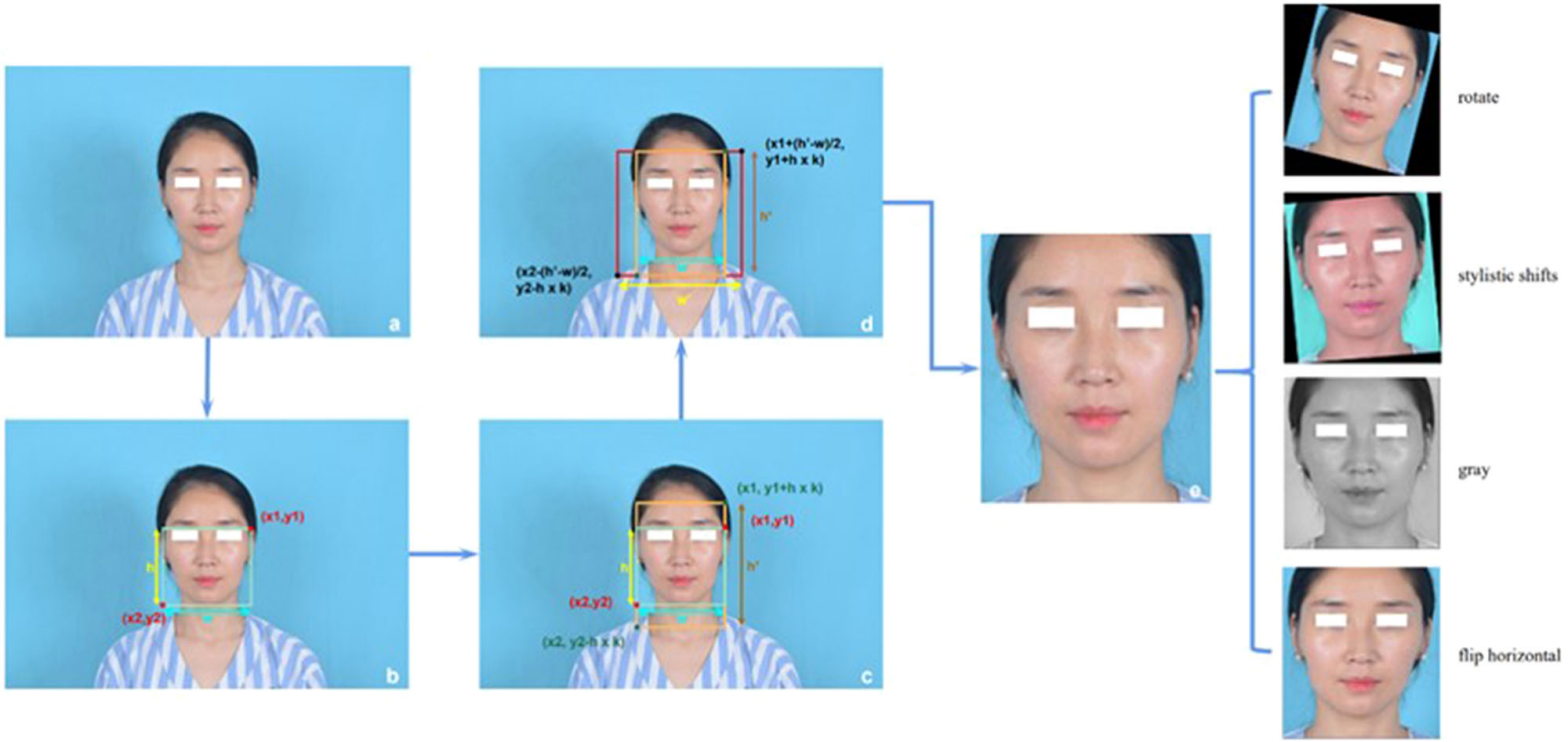
Image preprocessing and image enhancement. **a** Original image. **b** The coordinate values (x1, y1) and (x2, y2) were obtained, and the surface height h and surface width w were obtained by calculating y1-y2. **c** If h > w, for the upper boundary, the scale coefficient was set to k = 0.3, and the boundary was enlarged by y1+ (h × k). For the lower boundary, the scale coefficient was set to k = 0.1, and the boundary was enlarged by y2− (h × k). The height of the enlarged image was termed h̕. **d** To ensure the authenticity of the face aspect ratio, the aspect ratio of the cropped image should be 1:1, and the image boundary was expanded by x1− (h1 − w)/2 and ×2+ (h1 − w)/2. The expanded image width was termed w̕.

In practical applications, the obtained image may not be the same size as the training set images, and the aspect ratio of the input image may not meet the requirements of the network model. Therefore, in this study, artificial intelligence was fully utilized to process all the input images at a unified aspect ratio to meet the needs of neural networks in terms of the input image size. The resolution of the images taken by a conventional camera was 6000 × 4000. To balance the accuracy and computing speed of the DL model for predicting the key points of the face, the resolution of the image needed to be reduced to 256 × 256. For the training set images, the main body of the facial image was obtained by the maximum (x1) and minimum (x2) horizontal coordinates and the maximum (y1) and minimum (y2) longitudinal coordinates of the annotated marks. For the test set images, the application programming interface (API) in MediaPipe [30] was used for face detection to obtain the coordinate values (x1, y1) and (x2, y2), and the boundary range of the face was subsequently obtained, as shown in Fig. 3. The surface height (h) was obtained by calculating y1-y2, and the surface width (w) was obtained by calculating x1-x2. To guarantee that complete facial information was acquired, the boundaries had to be expanded. If h > w, for the upper boundary, the scale coefficient was set to k = 0.3, which enlarged the boundary by y1 + (h × k). For the lower boundary, the scale coefficient was set to k = 0.1, which enlarged the boundary by y2 − (h × k). The height of the enlarged image was set to h̕. To ensure the authenticity of the facial aspect ratio, the aspect ratio of the cropped image should be 1:1, and the image boundary was expanded by x1 + (h1 − w)/2 and ×2 − (h1 − w)/2. The expanded image width was w̕. The image was clipped using the above steps. Finally, Resize was used to adjust the resolution of the cropped image to 256 × 256.

After cropping, the image was enhanced through random operations such as rotation, mirroring, grayscale, and HSV format conversion during model training. The dataset was expanded 4 times to aid in generalized learning (Fig. 3).

### Heatmap principle of landmark recognition

In the landmark principle of Gaussian heatmap regression, the model regresses the heatmap at the pixel level and subsequently uses the predicted heatmap to infer the key point location. Using the opposite network structure to HR-Net, the input image is downsampled several times to obtain features of different sizes that are then fused; at the same time, upsampling ensures that the minimum size features are fully utilized to obtain richer semantic information.

This method pays more attention than other methods to local features. Furthermore, when the output feature map is large and the resolution is high, the landmarks predicted by this method are more accurate. The mathematical formula for generating a heatmap of the Gaussian kernel function is shown in (1).

1 $$f(x,y)={e}^{-\frac{{(x-{x}_{0})}^{2}+{(y-{y}_{0})}^{2}}{2{\sigma }^{2}}}$$

Here, ${{{{{\rm{\sigma }}}}}}$ is the Gaussian nuclear radius, the center of$\,{{{{{{\rm{x}}}}}}}_{0}$ is the Gaussian kernel abscissa, and ${{{{{{\rm{y}}}}}}}_{0}$ is the Gaussian kernel center ordinate.

**Fig. 4:**
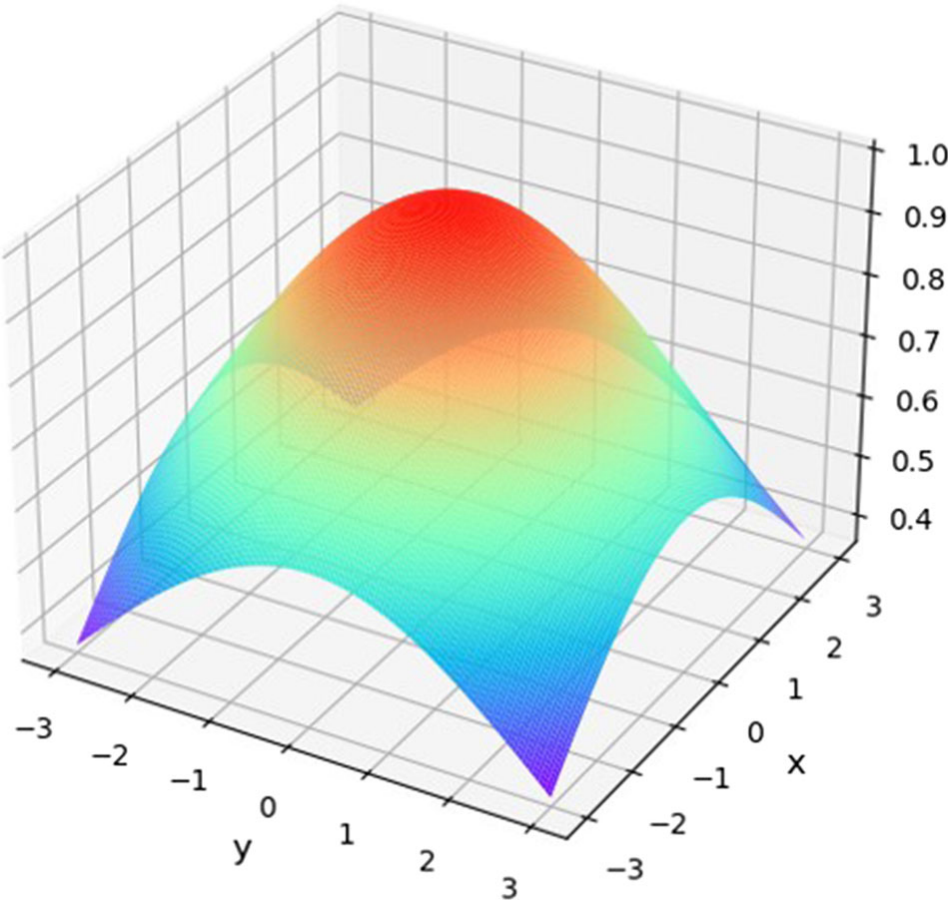
Schematic diagram of the Gaussian kernel.

The Gaussian kernel image is shown in Fig. 4. In the heatmap, the pixel value of a coordinate where a marker is located is 1, and the pixel value decreases outwards until it reaches 0. Due to the slow operation speed of regression based on heatmaps, the size of images is usually reduced. Since the resolution of the input image affects the prediction accuracy and operation speed [31], to balance the problems of speed and accuracy, the resolution of the input image is reduced to 1/4 of the original image in BHR-Net, and the number of pixels in the output image is set to 128*128 to reduce the normalization error of the model. Then, a heatmap is generated according to the size of the reduced image and the number and coordinates of the landmarks. The number of heatmaps is equal to the number of landmarks, and each heatmap corresponds to a key point coordinate.

### Architecture of BHR-Net

In this paper, we propose a simple and effective high-resolution back network that combines U-Net and HR-Net model structures to improve the semantic representation of high-resolution outputs. Before an image is input to BHR-Net, the resolution must be reduced to 1/4 of that of the original image to balance the prediction speed and prediction accuracy. The input image resolution is set to 256 × 256, and the output heatmap resolution of the network model is 64 × 64. In actual application, the resolution of the input image can be appropriately adjusted according to the number of soft tissue landmarks. For example, the resolution of the model output heatmap in the 34-point dataset is adjusted to 128 × 128, which significantly improves the prediction accuracy.

**Fig. 5:**
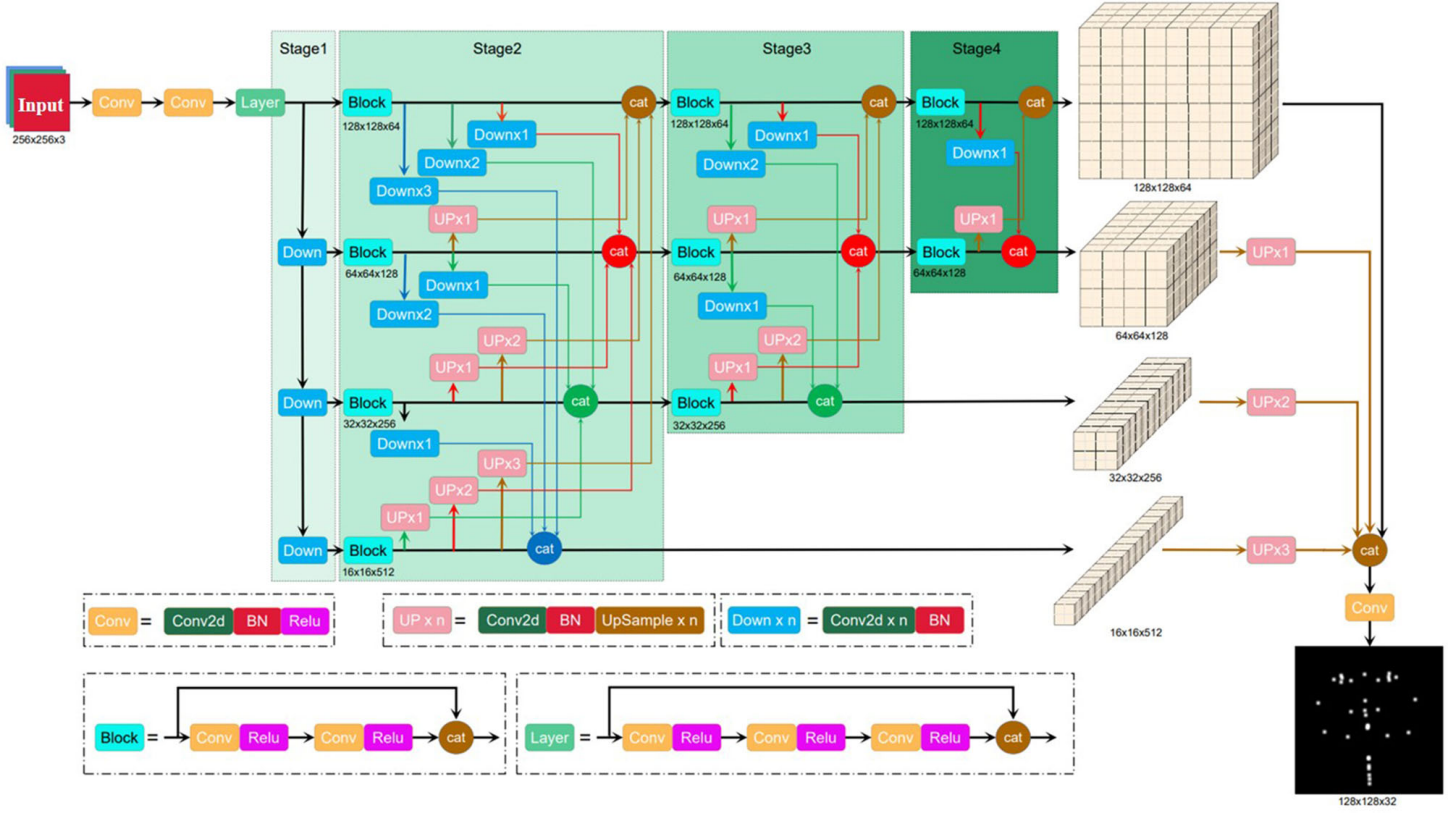
Architecture diagram of the Back High-Resolution Network outlining the deep learning model architecture. The architecture has 4 stages. Stage 1 obtains the branches of feature maps with different resolutions. Stage 2 to Stage 4 obtain and fuse multidimensional feature maps. Using the opposite network structure of HR-Net, the input image is downsampled several times to obtain features of different sizes, which are then fused; at the same time, upsampling ensures that the minimum size features are fully utilized to obtain richer semantic information.

The main body of the network model is divided into two parts, an encoder and a decoder. The encoder uses a method similar to U-Net to downsample the input features three consecutive times to obtain deeper features while saving the feature maps of different scales for skip connections with the corresponding feature maps in the decoder. The encoder also uses a convolution layer with a step size of 2 to replace the maximum pooling layer for downsampling (Stage 1). The decoder uses the HR-Net method to carry out feature fusion on all feature graphs of different scales. In our method, the number of features to be fused decreases at each stage. In Stage 2, feature graphs of all sizes that are output from the encoder are fused. The number of feature graphs is gradually reduced in Stage 2, Stage 3 and Stage 4, as only higher resolution feature maps are retained. Stage 4 inputs only the highest resolution feature maps. Finally, the lowest resolution feature maps of Stage 2, Stage 3 and Stage 4 are selected, and feature fusion is carried out with the output of Stage 4 to obtain the final output. This approach effectively improves the utilization rate of the feature graph with the lowest resolution and the strongest semantic feature and improves the prediction accuracy. To increase the depth of the network and extract deeper features, Stage 2 is repeated twice, and Stage 3 is repeated four times (Fig. 5).

### Improvements to BHR-Net

The smallest resolution feature map in HR-Net is output after only one fusion, although it often contains richer semantic information. The feature extraction method of BHR-Net constructed in this paper is the opposite of that of HR-Net. The upsampled low-resolution feature maps are fused with other high-resolution feature maps, and then features are extracted through a convolution operation until the highest feature map size is restored. In addition, the feature maps at each resolution other than the highest resolution are also output separately, and the feature maps at different resolutions are simultaneously upsampled to the same resolution as the highest feature map and fused. The final result is obtained through the convolution operation. In this way, even the smallest resolution feature map can be fully utilized.

### Loss function

This model uses the L2 loss function, also known as the mean squared error (MSE), which is commonly used in the field of key point detection. The average error between the predicted value and the actual value is evaluated by calculating the sum of squares of the distance between the predicted value and the actual value, and its range is 0 to +∞. The formula of the L2 loss function is shown in (2).

2 $${L}_{2}(Y,f(x))=\frac{1}{n}\mathop{\sum }\limits_{i=0}^{n}{({Y}_{i}-f{(x)}_{i})}^{2}$$

**Fig. 6:**
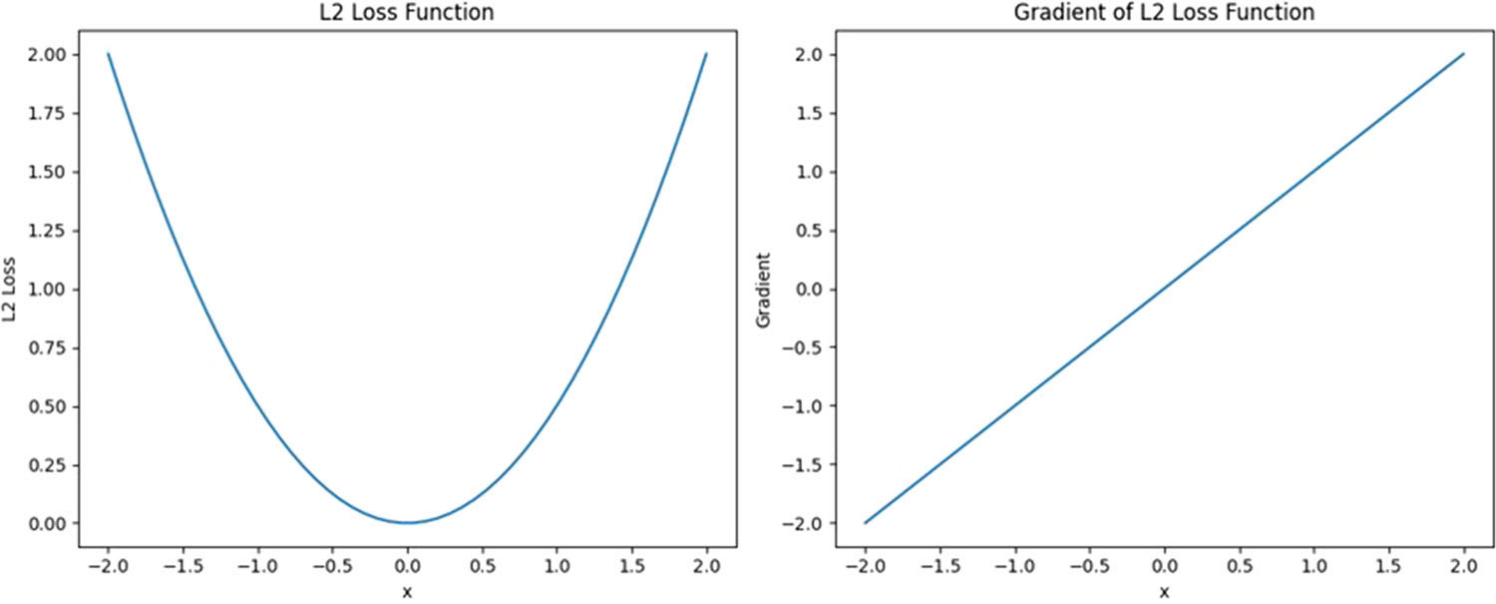
L2 loss function and its gradient diagram.

Where Y represents the predicted value, f(x) represents the true value, and n represents the number of key points. The gradient of the L2 loss function is x and is continuous at 0. The gradient is proportional to the size of the error; the larger the error is, the larger the gradient and the faster the convergence rate, and the smaller the error is, the smaller the gradient. However, the function is highly sensitive to outliers, large errors have too much influence on the direction of the gradient update, and the weights cannot be effectively updated if the errors are too small (Fig. 6).

### Model training

The network model used in this study is developed primarily based on the open-source HR-net framework model, and the PyTorch DL framework is used for training on the Linux system Ubuntu 20.04. The central processing unit (CPU) is an Intel (R) Xeon(R) Platinum 8358P (15 cores, 2.60 GHz). The graphics processing unit (GPU) is an NVIDIA GeForce RTX3090 (24G), and Compute Unified Device Architecture (CUDA) version 11.3 is used. All hyperparameter settings in the experiment are shown in Table 2.

**Table 2:** Experimental hyperparameter settings.

Optimizer	Adam
Initial learning rate	0.001
Learning rate decay strategy	Step beam attenuator
Learning rate attenuation frequency	Training 20 rounds of loss value does not decrease, the learning rate is reduced by 10 times
Submitted spec	16
Training rounds	300

**Fig. 7:**
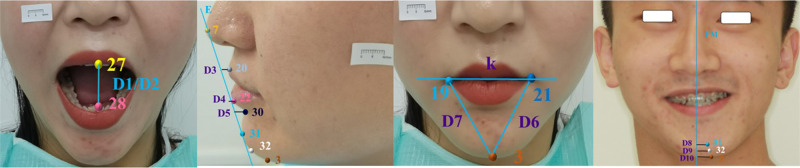
Schematic diagram of the distances between anatomical landmarks. 1. Facial esthetic line: The E line is composed of the line between Prn and Pog. 2. Facial midline (FM): the marker is on the facial midline; the points N, Prn, Sn, As, Ls and IIs are marked through the least square regression imaginary straight line. 3. In the plane Cartesian coordinate system, when the k value is positive, the confluence plane is inclined upwards; when the k value is negative, the confluence plane is inclined downwards; and when k = 0, the confluence plane is parallel to the horizontal plane. 4. The serial number of the marker points refers to the code of the marker points constituting the measurement index in each image shown in Fig. 2.

**Fig. 8:**
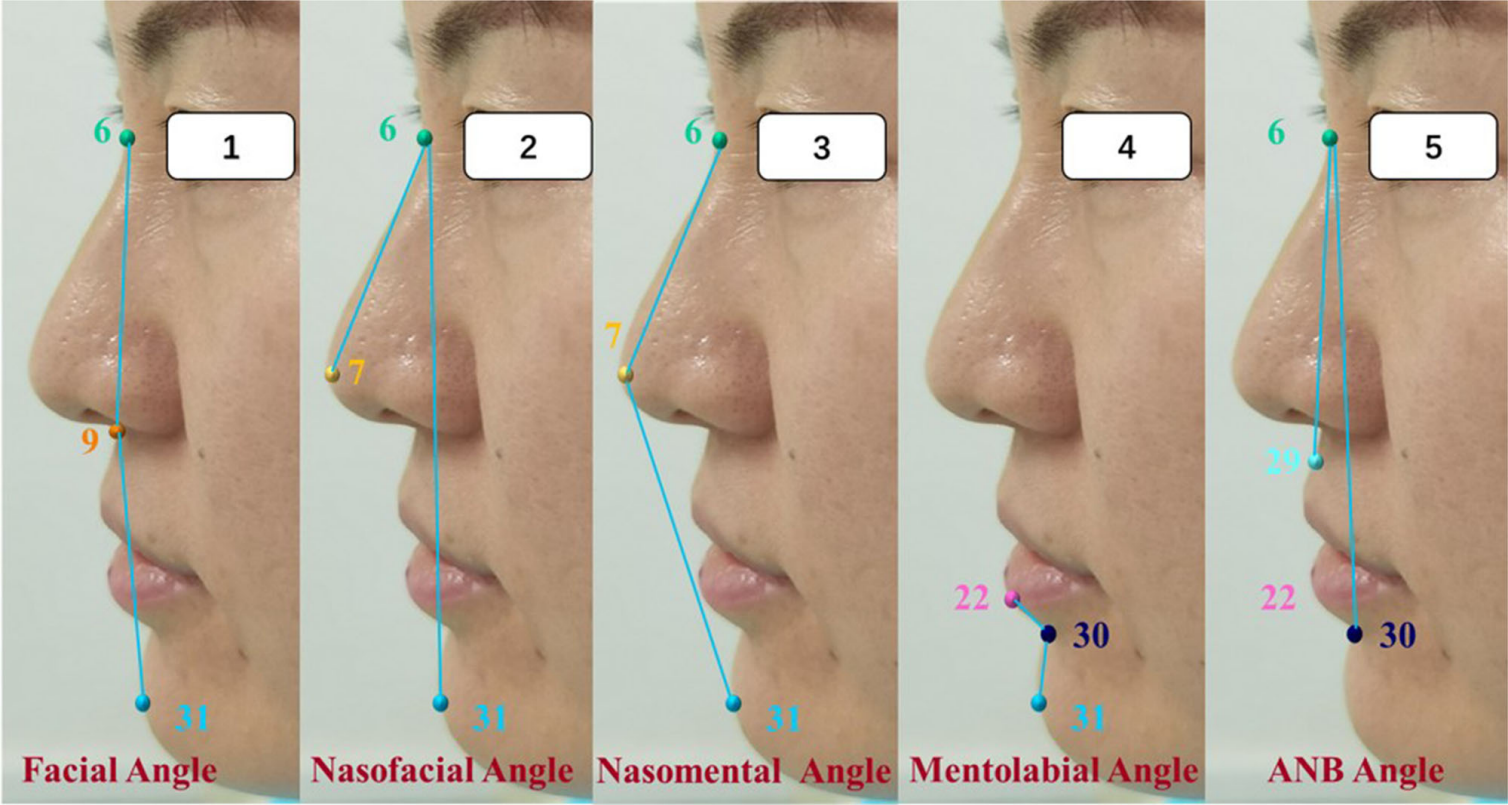
Angle diagram between anatomical landmarks.

First, before model training, the stability of the 3-observer labeled dataset is analyzed by the intragroup correlation coefficient (ICC). As shown in Fig. 2, 34 points are manually marked in the images of both the custom training set and the test set, including 32 anatomical points and “0” and “1” points. All 34 markers could be automatically recognized by BHR-Net. In this manuscript, 14 anatomic markers (Figs. 7 and 8) that constitute the measurement indicators for preoperative diagnosis of orthognathic surgery are selected, while the other 18 markers are not closely related to the topic of this paper.

Then, the preprocessed images are input into the BHR-Net model, and the average coordinate values of the open-source dataset and the manually annotated custom dataset are used for training. The Adam optimizer is used during training, the batch size is set to 16, and the initial learning rate is set to 0.001. When there are 20 training rounds and the loss value is no longer reduced, the learning rate is reduced tenfold to obtain the global optimal solution, and the training ends after 300 epochs.

### Performance evaluation indices and results

Interocular normalization (ION) aims to remove unreasonable changes due to inconsistencies in the dimensions of the face. The mathematical formula for ION is shown in (3):

3 $${{{{{{\rm{e}}}}}}}_{i}=\frac{{\Vert {x}_{pr{e}_{i}}-{x}_{g{t}_{i}}\Vert }_{2}}{{d}_{IOD}}$$

Here, ${x}_{{{pre}}_{i}}$ denotes the coordinate point prediction, and ${x}_{{{gt}}_{{i}}}$ denotes the real coordinate points. The subscripts$\,{x}_{i}$ are numbered one-to-one relative to the key points in Table 1. For example, in ION, ${d}_{{IOD}}=D\left(\left({x}_{36},{y}_{36}\right),\left({x}_{45},{y}_{45}\right)\right)$ indicates the outer canthal spacing between two eyes.

The mathematical formula of the mean normalized error (MNE) [32] is shown in (4). Here, ${x}_{{{pre}}_{i}}$ denotes the coordinate point prediction,$\,{x}_{{{gt}}_{i}}$ denotes the real coordinate points, ${d}_{{IOD}}$ denotes ION, and N is the number of key points. MNE represents the average error of N key point coordinates based on ION.

4 $$e=\frac{\mathop{\sum }\nolimits_{i=1}^{N}{\Vert {x}_{pr{e}_{i}}-{x}_{g{t}_{i}}\Vert }_{2}}{N\times {d}_{IOD}}\times 100 \%$$

For the failure rate (FR) [32] during sample prediction, if the normalized MSE is greater than 10%, then prediction failure is considered to have occurred. The proportion of the number of prediction failures in all samples to the total sample is expressed as the failure rate.

In this study, we tested BHR-Net on the WFLW and 300 W datasets and found that both MNE and FR improved. Moreover, the test results of BHR-Net improved by the heatmap regression method are obviously better than those of HR-Net on custom datasets (Table 3).

**Table 3:** Comparison of MNE and FR according to the experimental results for each model.

Index	Network model	WFLW	300 W			Customize
			Common	Challenging	Full	
MNE(%)	Resnet50	7.1	8.6	15.4	10.7	–
	Mobile-net	6.9	8.2	17.5	11.4	–
	DeCaFa [45]	6.6	–	–	–	–
	PIP-Net [18]	6.5	–	–	–	–
	EfficientNet-B3 [46]	7.8	–	–	–	–
	ATPN [47]	6.1	–	–	–	–
	3FabRec [48]	6.2	7.3	9.7	8.3	–
	HR-net	5.9	7.3	9.4	8.1	7.5
	BHR-net	**5.2**	**6.9**	**9.4**	**7.8**	**2.5**
FR > _10%_ (%)	Resnet50	11.9	10.9	20.6	11.6	–
	Mobile-net	14.35	10.2	29.4	17.3	–
	DeCaFa [45]	8.9	–	–	–	–
	PIP-Net [18]	8.2	–	–	–	–
	EfficientNet–B3 [46]	19.7	–	–	–	–
	ATPN [47]	7.4	–	–	–	–
	3FabRec [48]	8.2	–	–	–	–
	HR-net	7.1	8.6	16.3	12.1	7.2
	BHR-net	**6.9**	**7.8**	**13.7**	**10.6**	**1.4**

% omitted, – not counted.

The data in bold are the results of the BHR-net.

### Model testing and data analysis

BHR-Net was tested on a set of 50 human faces using the average value of manually marked data as the control group. The accuracy of BHR-Net in the recognition of landmarks was evaluated using the measurement indicators in Table 4. The statistical analysis software SAS was used to conduct a single-sample t test (the test standard was 2 mm) and a paired t test for measurement indicators.

**Table 4:** Measurements based on landmarks.

	No.	Measurements	Abbreviation	Marker number	Definition
Distance	1	D1 Large/D2 Slight	UI-LI	27-28	The distance of UI-LI
	2	D3	Ls-E	20	Horizontal distance from Ls to E
	3	D4	Li-E	22	Horizontal distance from Li to E
	4	D5	IIs-E	30	Horizontal distance from IIs to E
	5	D6 Left/D7 Right	C-Mes	19-32/21-32	The distance of C-Mes
	6	D8	Pog-FM	31	Horizontal distance from Pog to FM
	7	D9	Gn-FM	32	Horizontal distance from Gn to FM
	8	D10	Mes-FM	3	Horizontal distance from Mes to FM
Slope	1	K	CR-CL/x_0_	19/21	The slope of the bilateral Angle line to the horizontal plane
Angle	1	Facial angle	N-Sn-Pog	6-9-31	Angle between N-Sn and Sn-Pog
	2	Nasofacial angle	Prn-N-Pog	7-6-31	Angle between Prn-N and N-Pog
	3	Nasomental angle	N-Prn-Pog	6-7-31	Angle between N-Prn and Prn-Pog
	4	Mentolabial angle	Li-IIs-Pog	22-30-31	Angle between Li-IIs and IIs-Pog
	5	ANB angle	As-N-IIs	29-6-30	Angle between As-N and N-IIs

### Model application

Shahidi et al. [33] and Leonardi et al. [34] tested approximately 40 patients in their studies. In this study, after the model test was successful, facial anterior-lateral images of 30 patients with maxillofacial deformities diagnosed by experts were selected for application validation. The diagnosis was made by measuring indicators, and the accuracy was judged by a confusion matrix. Moreover, the preoperative and postoperative data of the AI group and the manual group were analyzed via paired t tests.

## Results

Multiple pose images are included in this study, and the markers that constitute the face measurement indicators in each pose image are not consistent. However, the DL algorithm requires the number of mark points in each pose to be consistent. Therefore, 34 points are marked and trained in this study, but statistical analysis is performed only for the landmark points constituting the measurement indices in any attitude image .

**Fig. 9:**
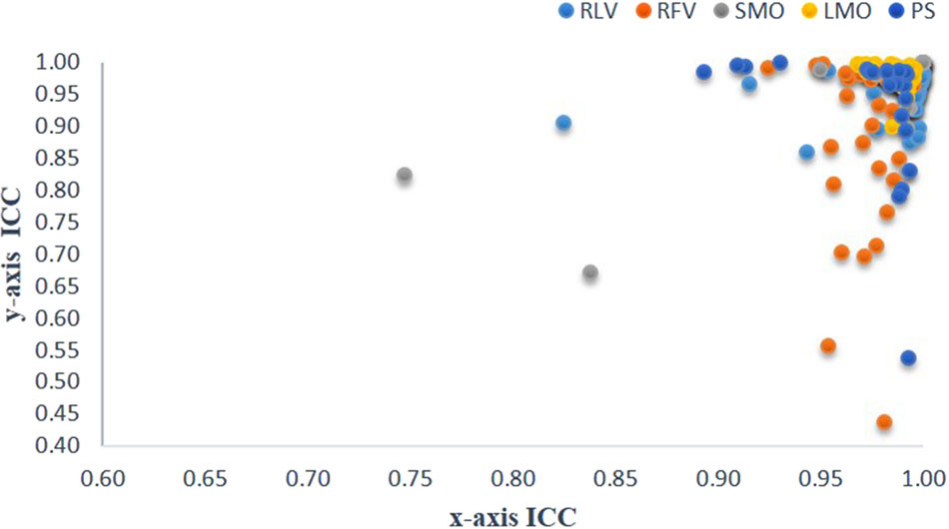
Statistical analysis of the intraobserver ICC distribution for 14 landmarks in the manual group.

**Fig. 10:**
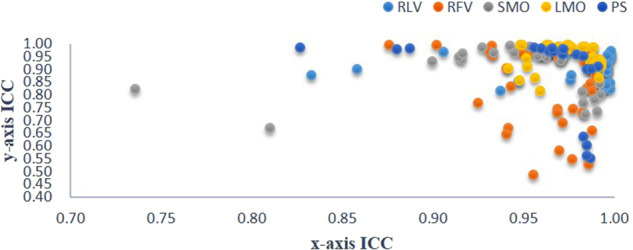
Statistical analysis of the interobserver ICC distribution for 14 markers in the manual group.

**Table 5:** Intraobserver ICCs of 14 landmarks in the manual group.

No	Name	observer	RFV		RLV		SMO		LMO		PS	
			x	y	x	y	x	y	x	y	x	y
1	Mes	A	0.92	0.99	0.92	0.97	0.97	0.99	0.98	1.00	0.93	1.00
		B	0.95	1.00	0.95	0.99	0.95	0.99	0.97	1.00	0.91	0.99
		C	0.95	1.00	0.82	0.91	1.00	1.00	0.97	0.99	0.91	1.00
2	N	A	0.98	0.44	1.00	0.96	0.99	0.83	0.99	0.97	0.99	0.80
		B	0.97	0.70	1.00	0.98	0.99	0.90	0.99	0.96	0.99	0.79
		C	0.98	0.77	1.00	0.93	1.00	1.00	0.99	0.90	0.99	0.54
3	Prn	A	0.99	0.91	1.00	0.96	0.99	0.93	0.99	0.96	0.99	0.89
		B	0.98	0.84	1.00	0.97	0.99	0.91	0.99	0.98	0.99	0.92
		C	0.99	0.85	1.00	0.90	1.00	1.00	0.99	0.95	0.99	0.83
4	Sn	A	0.98	0.93	1.00	0.95	0.99	0.93	0.99	0.97	0.99	0.97
		B	0.99	0.95	1.00	0.98	0.99	0.96	1.00	0.99	0.99	0.98
		C	0.99	0.98	0.99	0.87	1.00	1.00	1.00	0.98	0.99	0.96
5	CR	A	0.98	0.98	1.00	0.95	1.00	0.98	0.99	0.97	0.99	0.98
		B	0.97	0.97	–	–	0.99	0.96	1.00	0.98	0.99	0.98
		C	0.99	0.98	–	–	1.00	1.00	0.99	0.96	0.99	0.94
6	Ls	A	0.98	0.98	–	–	0.99	0.99	0.98	0.99	0.98	0.99
		B	0.99	0.97	1.00	0.98	0.99	0.97	0.99	0.99	0.99	0.98
		C	0.99	0.99	0.98	0.89	1.00	1.00	0.99	0.98	0.98	0.97
7	CL	A	0.97	0.98	–	–	1.00	0.98	0.99	0.96	0.99	0.99
		B	0.96	0.98	–	–	0.84	0.67	1.00	0.98	0.99	0.99
		C	0.99	0.99	–	–	1.00	1.00	0.99	0.97	0.99	0.97
8	Li	A	0.96	0.98	1.00	0.96	0.99	0.99	0.98	1.00	0.97	0.99
		B	0.98	0.98	1.00	0.98	0.75	0.82	0.97	1.00	0.97	0.99
		C	0.98	0.99	1.00	0.94	1.00	1.00	0.99	1.00	0.98	0.99
9	UI	A	–	–	–	–	0.98	0.98	0.97	0.98	0.98	0.98
		B	–	–	–	–	0.98	0.98	0.99	1.00	0.89	0.99
		C	–	–	–	–	1.00	1.00	0.99	0.99	0.98	0.96
10	LI	A	–	–	–	–	0.97	0.99	0.98	0.99	–	–
		B	–	–	–	–	0.99	0.99	0.99	1.00	–	–
		C	–	–	–	–	1.00	1.00	0.99	0.99	–	–
11	As	A	0.99	0.82	1.00	0.95	–	–	–	–	–	–
		B	0.98	0.92	1.00	0.97	–	–	–	–	–	–
		C	1.00	0.96	1.00	0.88	–	–	–	–	–	–
12	IIs	A	0.96	0.95	1.00	0.97	–	–	–	–	–	–
		B	0.98	0.90	1.00	0.98	–	–	–	–	–	–
		C	0.98	0.97	1.00	0.93	–	–	–	–	–	–
13	Pog	A	0.95	0.56	1.00	0.95	–	–	–	–	–	–
		B	0.96	0.70	1.00	0.98	–	–	–	–	–	–
		C	0.98	0.71	1.00	0.93	–	–	–	–	–	–
14	Gn	A	0.96	0.81	0.98	0.95	–	–	–	–	–	–
		B	0.96	0.87	0.99	0.98	–	–	–	–	–	–
		C	0.97	0.87	0.94	0.86	–	–	–	–	–	–

– not counted.

**Table 6:** Interobserver ICCs of 14 landmarks in the manual group.

No.	Name	Time	RFV		RLV		SMO		LMO		PS	
			x	y	x	y	x	y	x	y	x	y
1	Mes	1	0.93	0.99	0.91	0.97	0.95	0.99	0.96	1.00	0.83	0.98
		2	0.88	1.00	0.83	0.88	0.94	0.99	0.95	1.00	0.89	0.98
		3	0.90	0.99	0.86	0.90	0.93	0.99	0.95	1.00	0.88	0.98
2	N	1	0.97	0.69	1.00	0.96	0.99	0.73	0.99	0.90	0.99	0.55
		2	0.98	0.55	1.00	0.89	0.99	0.80	0.99	0.93	0.98	0.56
		3	0.97	0.58	1.00	0.91	0.99	0.78	0.99	0.95	0.99	0.60
3	Prn	1	0.98	0.82	1.00	0.94	0.98	0.72	0.99	0.92	0.98	0.64
		2	0.98	0.73	1.00	0.82	0.98	0.77	0.99	0.91	0.98	0.39
		3	0.99	0.66	1.00	0.85	0.98	0.81	0.99	0.87	0.98	0.33
4	Sn	1	0.98	0.93	1.00	0.95	0.99	0.89	0.99	0.94	0.99	0.91
		2	0.99	0.88	0.99	0.82	0.99	0.85	0.99	0.93	0.98	0.90
		3	0.99	0.84	0.99	0.85	0.99	0.84	0.99	0.91	0.99	0.90
5	CR	1	0.95	0.97	–	–	0.96	0.95	0.95	0.94	0.97	0.96
		2	0.93	0.95	–	–	0.96	0.95	0.94	0.90	0.97	0.97
		3	0.93	0.97	–	–	0.96	0.95	0.95	0.91	0.97	0.97
6	Ls	1	0.99	0.96	1.00	0.96	0.99	0.94	0.99	0.98	0.98	0.96
		2	0.99	0.93	0.98	0.86	0.99	0.94	0.99	0.98	0.97	0.98
		3	0.98	0.95	0.98	0.88	0.99	0.95	0.97	0.99	0.97	0.98
7	CL	1	0.95	0.97	–	–	0.97	0.94	0.96	0.87	0.98	0.95
		2	0.97	0.95	–	–	0.97	0.93	0.95	0.86	0.96	0.97
		3	0.96	0.96	–	–	0.81	0.67	0.96	0.82	0.97	0.97
8	Li	1	0.96	0.98	1.00	0.96	0.97	0.99	0.97	1.00	0.96	0.98
		2	0.97	0.97	1.00	0.90	0.97	0.99	0.97	1.00	0.96	0.98
		3	0.97	0.98	1.00	0.91	0.74	0.82	0.96	0.99	0.97	0.98
9	UI	1	–	–	–	–	0.90	0.93	0.98	0.98	0.51	0.94
		2	–	–	–	–	0.92	0.94	0.98	0.99	0.49	0.97
		3	–	–	–	–	0.91	0.95	0.97	0.99	0.52	0.96
10	LI	1	–	–	–	–	0.92	0.96	0.97	0.98	–	–
		2	–	–	–	–	0.93	0.97	0.98	0.98	–	–
		3	–	–	–	–	0.95	0.96	0.98	0.99	–	–
11	As	1	0.99	0.85	1.00	0.95	–	–	–	–	–	–
		2	0.99	0.82	1.00	0.83	–	–	–	–	–	–
		3	0.99	0.53	1.00	0.84	–	–	–	–	–	–
12	IIs	1	0.97	0.75	1.00	0.97	–	–	–	–	–	–
		2	0.98	0.75	1.00	0.89	–	–	–	–	–	–
		3	0.97	0.73	1.00	0.91	–	–	–	–	–	–
13	Pog	1	0.94	0.67	1.00	0.95	–	–	–	–	–	–
		2	0.96	0.49	1.00	0.89	–	–	–	–	–	–
		3	0.94	0.65	1.00	0.92	–	–	–	–	–	–
14	Gn	1	0.94	0.90	0.97	0.95	–	–	–	–	–	–
		2	0.94	0.83	0.94	0.82	–	–	–	–	–	–
		3	0.92	0.77	0.95	0.85	–	–	–	–	–	–

– not counted.

### Stability of manually labeled data (Tables 5 and 6, Figs. 9 and 10)

The following intraobserver ICCs were <0.75: Pog, N point in RFV, N point in PS, and intraobserver Y axis of CL point in SMO. The ICCs of the axis of other landmarks are ≥0.75.

The coordinate axes with an ICC < 0.75 between observers are Li in SMO and UI and X-axis in PS; the N-point, Prn, As, IIs, and Pog in RFV; Y-axis in Mes, N, and Prn in SMO; and CL in PS. All other ICCs are ≥0.75.

**Fig. 11:**
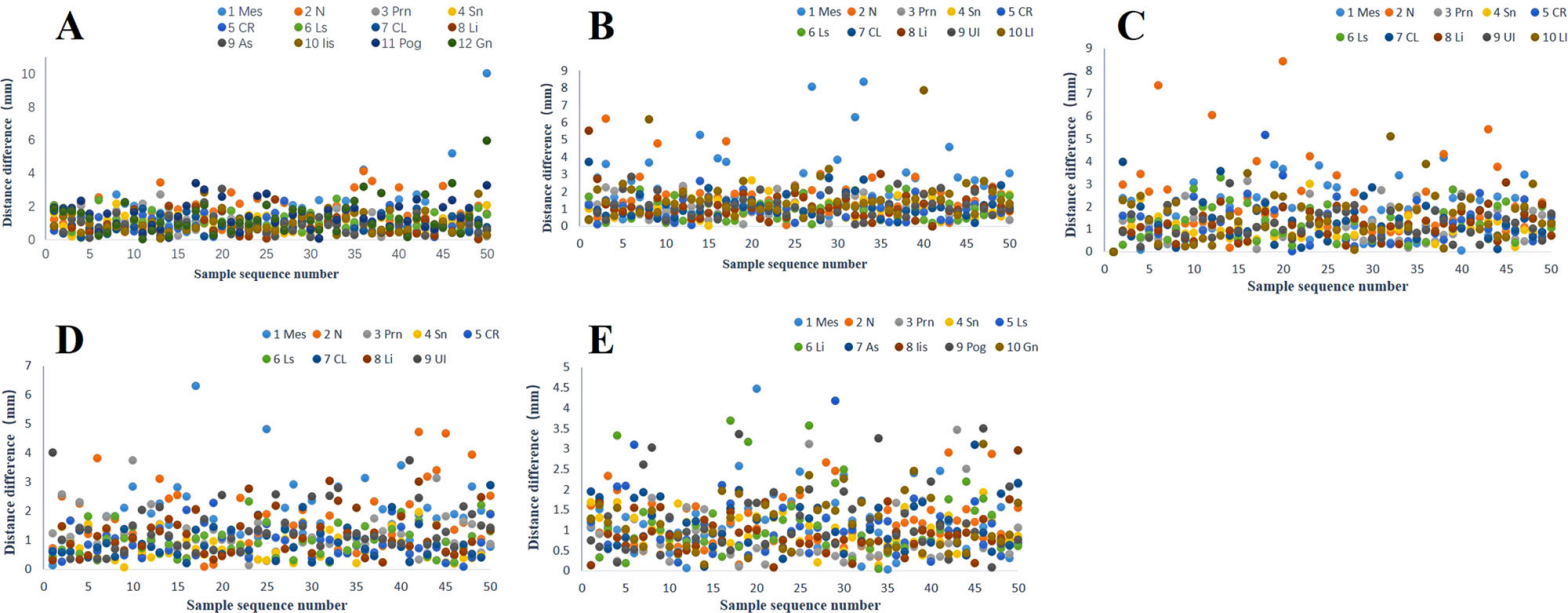
Statistical graph of error values of landmarks. **A** RFV, **B** SMO, **C** LMO, **D** PS, **E** RLV.

**Table 7:** Mean distance, standard deviation, percentage of ≤2 mm mark points and percentage of ≤4 mm mark points between the AI and manual groups in multipose images.

No.	Abb	RFV				SMO				LMO				PS				RLV			
		M	SD	2 mm	4 mm	M	SD	2 mm	4 mm	M	SD	2 mm	4 mm	M	SD	2 mm	4 mm	M	SD	2 mm	4 mm
1	Mes	1.68	1.54	72	94	2.36	1.75	58	100	1.75	1.05	64	100	1.78	1.12	66	96	1.24	0.79	86	98
2	N	1.59	0.93	70	98	1.69	1.15	76	94	2.25	1.71	60	86	1.67	1.13	66	96	1.21	0.65	90	100
3	Prn	1.06	0.6	92	100	1.05	0.55	90	100	1.27	0.73	84	100	1.21	0.69	90	100	0.84	0.72	94	100
4	Sn	0.94	0.44	96	100	1.06	0.52	96	100	1.1	0.55	94	100	0.79	0.48	100	100	0.93	0.41	100	100
5	CR	0.97	0.48	96	100	1.04	0.62	94	100	1.35	0.92	82	100	0.95	0.51	98	100	‒	‒	‒	‒
6	Ls	1.17	0.56	92	100	1.03	0.63	88	100	1.2	0.75	82	100	0.98	0.53	96	100	1.12	0.72	90	98
7	CL	0.94	0.49	100	100	1.2	0.71	86	100	1.32	0.8	82	100	0.92	0.55	94	100	‒	‒	‒	‒
8	Li	0.92	0.54	96	100	1.2	0.88	90	98	1.2	0.58	90	100	1.22	0.71	84	100	1.17	0.85	86	100
9	UI	‒	‒	‒	‒	1.17	0.62	86	100	1.18	0.61	92	100	1.42	0.81	78	98	‒	‒	‒	‒
10	LI	‒	‒	‒	‒	1.71	1.29	74	96	1.6	1	68	98	‒	‒	‒	‒	‒	‒	‒	‒
11	As	0.88	0.56	96	100	‒	‒	‒	‒	‒	‒	‒	‒	‒	‒	‒	‒	1.13	0.59	96	100
12	IIs	1.02	0.6	96	100	‒	‒	‒	‒	‒	‒	‒	‒	‒	‒	‒	‒	0.91	0.5	98	100
13	Pog	1.5	0.82	78	100	‒	‒	‒	‒	‒	‒	‒	‒	‒	‒	‒	‒	1.24	0.82	86	100
14	Gn	1.35	1.03	82	98	‒	‒	‒	‒	‒	‒	‒	‒	‒	‒	‒	‒	1.18	0.65	92	100

Percentage of landmarks with an error ≤2 mm or ≤4 mm in the 50 test images.

*Abb* Abbreviations, *M* mean, *SD* standard deviation, – not counted.

### Accuracy of landmark recognition (Table 7, Fig. 11)

In the test set of 50 patients, the average values of the predicted marker points are compared with those marked by the manual group. 1. The accuracy of the submental points in the RFV, SMO, LMO and PS poses is low (*p* > 0.05), and the 95% confidence interval contains 0; therefore, these differences cannot be rejected. 2. The accuracy of the nasal root point in the SMO and LMO postures is low (*p* > 0.05), and the 95% confidence interval contains 0; therefore, the difference cannot be rejected. 3. In RLV, 3 cases of error exist at the Prn point, and 1 case of error exists at the Sn point; these cases should be eliminated from the statistical analysis. The landmark accuracy in all the other pose images is very high (*p* = 0), the 95% confidence interval does not contain 0, and the difference can clearly be rejected. 4. When the error standard is controlled to 2 mm, the Mes point and N point have the lowest proportions in each pose. 5. When the error standard is controlled to 4 mm, the proportion of points marked <4 mm in all the images is as high as 94%, except for the nose root point of the LMO images, which is 86%.

**Fig. 12:**
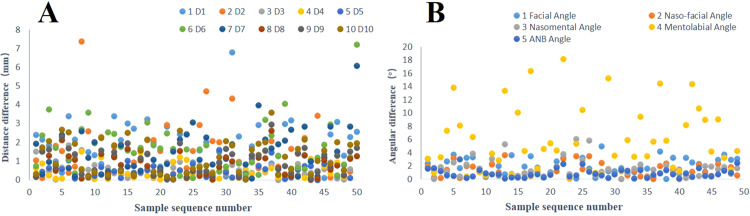
Statistical analysis of the difference between the AI and manual groups in the test set. **A** Distance. **B** Angle.

**Table 8:** Single sample t test results of the distance measurement indices of BHR-Net and the manual group.

	No.	Abb	BHR-Net				Manual group			
			M	SD	95% confidence interval		M	SD	95% confidence interval	
					Lower limit	Upper limit			Lower limit	Upper limit
Distance (mm)	1	D1	39.25	7.19	37.19	41.31	38.32	7.08	36.29	40.36
	2	D2	14.89	5.21	13.4	16.39	15.38	5.35	13.84	16.92
	3	D3	2.25	1.73	1.75	2.74	2.38	1.68	1.90	2.86
	4	D4	1.72	1.24	1.37	2.08	1.67	1.35	1.28	2.05
	5	D5	3.89	1.39	3.49	4.29	3.73	1.40	3.33	4.13
	6	D6	52.00	4.62	50.69	53.32	52.6	4.54	51.31	53.89
	7	D7	52.08	4.47	50.81	53.34	52.17	4.52	50.88	53.45
	8	D8	1.43	1.15	1.10	1.76	0.97	0.81	0.74	1.20
	9	D9	1.78	1.34	1.40	2.16	0.85	0.64	0.67	1.04
	10	D10	2.34	1.69	1.88	2.84	1.13	0.81	0.90	1.36
Slope	11		0	0.03	−0.01	0.01	0	0.03	−0.01	0

**Table 9:** Mean, standard deviation, 95% confidence interval and correlation coefficient of the slope and distance difference in the test set.

	No.	Abb	M	SD	95% confidence interval		*P*
					Lower limit	Upper limit	
Distance (mm)	1	D1	0.77	2.03	0.2	1.35	0.01
	2	D2	−0.48	1.67	−0.96	−0.1	0.05
	3	D3	−0.13	0.69	−0.33	0.06	0.18
	4	D4	0.06	0.63	−0.13	0.24	0.55
	5	D5	0.16	0.59	−0.01	0.32	0.06
	6	D6	−0.59	2.02	−1.17	−0.02	0.05
	7	D7	−0.08	1.88	−0.62	−0.45	0.76
	8	D8	0.46	1.44	0.48	0.87	0.29
	9	D9	0.93	0.71	0.72	1.13	<0.01
	10	D10	1.22	0.89	0.97	1.48	<0.01
Slope	11		0	0.02	0	0.01	0.59

**Table 10:** Results of single sample t tests of the angle measurement indices of BHR-Net and the manual group.

	No.	Measurements	BHR-Net				Manual group			
			M	SD	95% confidence interval		M	SD	95% confidence interval	
					Lower limit	Upper limit			Lower limit	Upper limit
Angle (°)	1	Facial angle	163.62	5.97	161.90	165.34	163.60	7.05	161.68	165.63
	2	Nasofacial angle	28.39	3.62	27.35	29.43	28.59	3.84	27.49	29.70
	3	Nasomental angle	133.39	5.86	131.71	135.07	133.07	5.98	131.36	134.79
	4	Mentolabial angle	136.40	9.88	133.57	139.24	139.21	7.70	137.01	141.43
	5	ANB angle	7.74	2.38	7.05	8.42	7.69	2.55	6.95	8.42

**Table 11:** Mean value, standard deviation and *P* value of each angle difference in the test set.

	No.	Measurements	M	SD	95% confidence interval		*P*
					Lower limit	Upper limit	
Angle (°)	1	Facial angle	0.02	2.35	−0.65	0.7	0.95
	2	Nasofacial angle	−0.21	1.61	−0.67	0.26	0.37
	3	Nasomental angle	0.31	2.13	−0.28	0.93	0.31
	4	Mentolabial angle	−2.81	7.05	−4.84	−0.79	0.01
	5	ANB angle	0.05	1.12	−0.27	0.37	0.75

### Accuracy of the test indicators (Tables 8–11, Fig. 12)

In the 50-image test set, the measurement indices of the predicted markers are compared with those of the mean coordinate values of manually labeled markers: the *p* values of D2, D3, D4, D5, D6, D7, D8 and k are ≥0.05, and no significant difference exists. The *p* values for D1, D9 and D10 are 0, indicating a statistically significant difference.

After comparison of facial angles, the *p* values of the facial angle, nasofacial angle, nasomental angle and ANB angle are all ≥0.05, and the correlation coefficients are all ≥0.9, indicating no significant differences. The *p* value of the difference in the mentolabial angle is <0.05, which indicates a significant difference.

### Confusion matrix

Thirty patients were examined—10 patients each with Class II or Class III deformities or MADs. The diagnostic accuracy for Class II and III deformities is 100%. The classification and diagnostic accuracy of MADs is 70%. The classification and diagnostic accuracy of the occlusal plane is 100% (Tables 12 and 13).

**Table 12:** Confusion matrix results for the classification and diagnosis of bone malocclusion.

	Prediction					
	II	III		Left skew	Right skew	
Actual	II	10	0	Left skew	4	1
	III	0	10	Right skew	2	3

Evaluation criteria: 1. Face angle ≤157° for Class II. ≥165° for Class III. 2. The distance between point C on both sides and Mes is determined.

**Table 13:** Confusion matrix results of occlusal plane classification diagnosis for MAD.

		Prediction		
		−k	k = 0	+k
Actual	−k	5	0	0
	k = 0	0	2	0
	+k	0	0	3

Evaluation criteria: In the plane rectangular coordinate system, when the value of k is positive, the occlusal plane is oblique upwards. When the k value is negative, the occlusal plane is oblique downwards. At k = 0, the occlusal plane is parallel to the horizontal plane.

The preoperative and postoperative effects in 30 patients were assessed by paired t tests between the AI and manual groups. The *p* value for patients with Type II and Type III bone malocclusion in the AI group is 0, indicating significant differences. The *p* value for patients with mandibular deformity in the AI group is 0.26, which is not significantly different, and the *p* value for patients with mandibular deformity in the manual group is 0.93. A comparison indicated that the results of the AI group are consistent with those of the manual group (Table 14).

**Table 14:** Preoperative and postoperative comparison results of 20 patients with Class II and Class III malocclusion and 10 patients with MAD analyzed by AI and manual analysis.

	AI					Manual group				
	M	SD	95% confidence interval		*P*	M	SD	95% confidence interval		*P*
			Lower limit	Upper limit				Lower limit	Upper limit	
II (°)	−7.2	3.04	−9.4	−5.06	0	−4.47	2.84	−6.3	−2.24	0
III (°)	7.8	1.31	4.87	10.78	0	4.87	3.98	2.02	7.71	0
MAD (mm)	−1.99	5.22	−5.73	1.74	0.26	0.65	2.41	−1.66	1.79	0.93

## Discussion

The aim of this study is to construct a network model that can automatically acquire facial features and provide diagnostic information for personalized diagnosis and treatment rather than building a database of average faces. Therefore, 34 markers are labeled for training purposes, focusing on the accuracy of 16 markers (including scale markers 0 and 1) that are closely related to orthognathic surgery diagnosis. This is a strength of this study. Through the introduction of a scale, the detection results of BHR-Net can be applied to clinical work to assist clinicians in diagnostic analysis. The other 18 markers have low correlations with the disease types studied in this paper, and the findings with these markers will be published in a separate paper due to space constraints. In repetitive inspection work, the network model successfully constructed in this paper can effectively avoid background dependence of manual measurement and reduce measurement error [35].

Soft tissue measurements are important components of cephalometric measurements and are highly important for the diagnosis and analysis of orthognathic surgery cases and for the design of corrections [28]. The use of the soft tissue concept in orthognathic treatment has become a topic of interest [29]. As shown in Table 4, in clinical practice, the mouth opening is the distance between two UI-LI points. The line between the Prn and Pog marks represents Rickett’s E line. The horizontal distance from Ls, Li and IIs to the E line can determine the relationship between the nose, lip and chin. The distance between points C and Me on both sides is used to judge the degree of deflection of the chin. The slope k formed by the line at point C on both sides and the horizontal line can indicate whether the occlusal plane is horizontal. Landmarks such as N, Sn, Pog, Prn, Li, IIs, and AS constitute corresponding measurement angles to judge the degree of facial soft tissue deformity [29] (Figs. 7 and 8). The values measured between these landmarks are reference indices for clinicians during diagnosis and are important for accurately evaluating the postoperative outcome of orthognathic surgery. Therefore, the identification of anatomic landmarks quickly and accurately is worth studying. However, in clinical practice, due to the inconsistent positioning of markers, the measurement of indicators between markers is complicated, which may lead to large differences between the values measured by each doctor, and the results are unreliable.

The application of AI in orthognathic surgery has been widely studied, and researchers have been committed to studying automatic mark recognition to reduce the time needed for cephalometric analysis and to improve recognition accuracy. Ye-Hyun Kim compared the depth and structure of different network models in determining whether orthognathic surgery is needed and reported that ResNet-18 had the best results [36]. Ji-Hoon Park and Hye-Won Hwang identified radiograph markers by comparing You-Only Look-Once version 3 (YOLOv3) and the single shot multibox detector (SSD). YOLOv3 was shown to have better accuracy than the other methods [37, 38]. Yao J et al suggested that these results are accurate for the automatic recognition of landmarks when the error is less than 2 mm and that the results are acceptable when the error is less than 4 mm [39]. Shahidi S identified 16 landmarks on 40 skull radiographs with an average error of 2.59 mm [33], and Leonardi R identified 10 landmarks on 41 radiographs [34]. Based on facial soft tissue images, Jeong SH used the Visual Geometry Group 19 (VGG19) network model to recognize facial soft tissue images with an accuracy of 89.3% [12]; however, VGG consumed more memory and occupied more computing resources than the other models. Recent studies all have certain limitations, such as high operational costs, few training sets, and few measurements [40]. The results of this study show that among the 14 markers identified via statistical analysis, when the standard error is 4 mm, the accuracy of all the markers is as high as 94%, except for the N point of the LMO image, for which the accuracy is 86%. When the standard error is 2 mm, the accuracy of Pog, Li and Mes on lateral images is 86%, and the accuracy of the other landmarks is greater than 90%. On the other hand, the accuracy of Mes, N, Pog and Li on frontal images, including RFV/SMO/LMO/PS, is low, which may be related to the flat anatomical position, which is not conducive to BHR-Net detection. For these landmarks, the next step is to apply the latest network model proposed by Wan et al. [26] and Kang et al. [27] to improve the accuracy of the detection results.

There are many models that implement facial feature detection, such as Google MediaPipe [30], Face++ [41], and Baidu [42, 43]. In contrast, the model in this study was designed according to clinical diagnostic requirements, and the 32 markers selected were clearly defined anatomically and may not be fully included in the 68-point 300 W model or the 98-point WFLW model. Therefore, in this study, a custom dataset applicable to BHR-Net was constructed, and BHR-Net was compared with existing models (Table 3). The NMS of BHR-Net on the WFLW dataset is 5.2%, and the failure rate is 6.9%. For the 300 W dataset, the common test result is 6.9%, the challenge test result is 9.4%, the full test result is 7.8%, and the custom dataset NMS result is only 2.5%. The failure rate of BHR-Net is also the lowest of all the models, and the failure rate of the custom dataset is only 1.4%. However, compared with the detection results of 300 W and WFLW based on the latest heatmap regressions of Wan et al. [26] and Kang et al. [27], there is still a gap. This study can learn from their network model for further research.

When a facial image has a large posture, heavy occlusion and complex illumination, most facial landmark detection methods cannot learn the discriminant feature representation and effective facial shape constraints or accurately predict the value of each element in the landmark heatmap, limiting their detection accuracy [26]. Therefore, when constructing a custom dataset, this study first adopts a data enhancement method to adjust the training set image through four methods: “rotate”, “stylistic shifts”, “graying” and “horizontal flipping”. Second, to avoid different image recognition effects of different qualities, the number of pixels in the input image is set to only 256 × 256, which is the capability that can be achieved by the current camera equipment. Third, the backgrounds of the custom training set images collected in this study are white or blue, without interference or other scenes. Therefore, after the training of BHR-Net in this study, only the rear camera of the mobile phone is used to obtain the input image; moreover, professional equipment and places are not required for image acquisition, as only the input image needs to meet the acquisition requirements. Then, through automatic cropping of the facial image, a uniform size of the input image can be obtained by adjusting the height-to-width ratio even if the size of the input image is different. In this study, in both the LMO and SMO images, there is 1 case of marker recognition error due to the lower central incisor teeth being unexposed. In LMO, N-point displacement occurs at the maximum opening in some volunteers’ images, which increases the difficulty of recognition. Therefore, to ensure more accurate results from the mouth opening test, individuals should avoid looking up, and the middle and upper parts of the face should be kept relatively static. The camera should be perpendicular to the opening plane.

At present, there are few applied studies on the measurement of multipose facial soft tissue images by neural networks. Most related research has been limited to automatic landmark recognition, and further accuracy analysis of additional measurement indicators has been insufficient [37–39]. Among the 14 markers analyzed in this study, the accuracy of Mes, N and Pog in RFV is low. This difficulty is related to the difficulty of AI recognition caused by the fact that these three markers are in a facial area that has a large radian or is relatively flat. The stability analysis of the manual group showed the same result. The results of manual labeling showed that the horizontal position of the landmarks in the middle of the face is relatively easy to determine, while stability in the vertical direction is relatively poor. Therefore, to reduce bias in system training during the construction of the training set, the average value of 9 annotations can be used as the training set, but the early labeling work is very large. Among the 10 distance indicators and 5 angle indicators calculated in this study, the distances from Pog to the midline of the plane and to the mentolabial angle are relatively poor due to the difficulty and low accuracy of Pog recognition, while the other measurement indicators all achieved the expected effect. By continuously improving the accuracy of marker recognition, better prediction results can be obtained. Expanding the number of training sets to ensure that the model obtains more training data is the most effective way to improve the accuracy of marker recognition.

Through a retrospective study of the case data of 30 patients with malformations, this model showed that based on the anatomical markers identified by BHR-Net, clinicians can objectively obtain the values of the measurement indicators, which can aid in the diagnosis and analysis of Class II and III patients. The preoperative and postoperative measurements were significantly different, and the results were credible. This is because all patients in this group underwent bilateral sagittal split osteotomy (BSSO), and mandibular movement significantly changed the mandibular profile [44].

Several studies have suggested that the mandibular contour has the greatest influence on facial symmetry [13]; therefore, in the present study, we focused on the changes in the mandible and occlusal plane in MAD patients. The slope k of the line at point C on both sides was used to evaluate the difference in roll direction, and the diagnostic accuracy reached 100%. However, when the distance between point C and point Mes on both sides was used to evaluate the difference in yaw direction, the results exhibited no significant difference, and the accuracy was only 70%. However, these results do not indicate poor performance of the proposed network model. First, only 10 MAD patients were analyzed in this study, resulting in an overly small sample size. Second, after the bone tissue is restored to normal after MAD surgery, the shape of the soft tissue still causes facial asymmetry in some patients after surgery. These results suggest that doctors should fully consider the influence of soft tissue when planning mandibular surgery. Correcting only the symmetry of hard tissue cannot completely address facial asymmetry in patients.

This study has four distinct advantages. First, highly professional custom image datasets suitable for orthognathic surgery were successfully collected, including 1030 maxillofacial developmental deformity images and 1183 facial multipose images; the training set of this study contained professional and diverse images. Second, the stability of the 3-person annotated data was first proven through ICC analysis, and the average coordinate values of the 3 independent annotated coordinate values were subsequently obtained to construct the training set and test set. This method can avoid system error and test set measurement bias caused by the single-person annotated training set. Third, the BHR-Net model constructed in this paper has strong generalizability. The network achieves accurate recognition and application of multipose facial image landmarks and provides a reference for rapid measurement and diagnosis in orthognathic surgery. Finally, a scale is innovatively added to the facial image, which enables the calculation of not only the angle between the landmarks but also the real distance.

It is undeniable that the development trend of 2D models is 3D, and the research basis of 3D models is 2D, which is why we chose 2D images. Our future research direction will focus on 3D models and the realization of automatic model diagnosis.

## Limitations

Although this study achieved some satisfactory results, there are still several shortcomings. First, the number of training set samples is insufficient, resulting in insufficiently accurate training results for some landmarks. Second, the background color and posture of the customized training set images are not rich, so the background color of the input image needs to be consistent with or similar to the background color of the training set. Third, the performance of the computing equipment is not strong enough, resulting in insufficient resolution of the input and output images. Fourth, the number of patients included for the validation of the model was insufficient, and additional clinical cases should be collected to verify the accuracy of the model. Finally, no further framework has been proposed for the diagnosis of facial deformities. The diagnosis must eventually be made manually by clinical doctors.

## Conclusions

In this study, a network model based on heatmap regression is successfully developed. The powerful spatial generalization ability of the model allows it to effectively identify the landmarks in maxillofacial multipose images and objectively and rapidly evaluate the deformities of facial features to accurately diagnose those deformities. As a result, a rapid and objective tool for measuring soft tissue topography in clinical practice was successfully developed in this work.

## Data Availability

The code for this model is available at https://github.com/zhougui?tab=repositories. The custom datasets in this study may be made available upon reasonable request by the reader by contacting the corresponding author and signing a confidentiality agreement with permission. Corresponding author’s email: MQ18710966911@163.com.
